# An Inquest on the Body of Harry Westley Boyle Haines

**Published:** 1899-07

**Authors:** 


An inquest was recently held by Mr. Troutbeck on the body of Harry Westley Boyle Haines, aged nineteen, a private in the Royal Berkshire Regiment, who died in St. George's Hospital. The evidence of the father, a garrison sergeant-major, with three sons in the army, showed that on December 17th last, when stationed at Portsmouth with his regiment, the deceased had a violent fit of coughing and swallowed a false tooth and plate. Efforts were made to extract the tooth from the throat, but these failed, and the tooth was, therefore, pushed down. The father had him brought up to St. George's Hospital. There, by means of the Routgen rays, the tooth was detected in the lad's throat, and an operation was performed, the tooth and plate being extracted with apparent success. The deceased improved steadily, but then became worse and died somewhat suddenly. The house surgeon stated that a post-mortem examination showed that there was a clot of blood in the jugular vein. This clot was septic, and had set up inflammation of the coating of the vein. The jury returned a verdict of accidental death. This recruit would evidently appear to have had defective teeth. The result is a sad one for his family and himself and a loss to the country, for the expense of shaping the recruit into the finished soldier is no small matter.—*The Dental Record.*

